# Non-Steroidal Anti-Inflammatory Drug Effect on the Binding of Plasma Protein with Antibiotic Drug Ceftazidime: Spectroscopic and In Silico Investigation

**DOI:** 10.3390/ijms241914811

**Published:** 2023-09-30

**Authors:** Mohd Sajid Ali, Ekampreet Singh, Jayaraman Muthukumaran, Hamad A. Al-Lohedan

**Affiliations:** 1 Surfactant Research Chair, Department of Chemistry, College of Science, King Saud University, P.O. Box 2455, Riyadh 11451, Saudi Arabia; hlohedan@ksu.edu.sa; 2 Department of Biotechnology, Sharda School of Engineering and Technology, Sharda University, Greater Noida 201310, India; ekampreetsingh024@gmail.com (E.S.); j.muthukumaran@sharda.ac.in (J.M.)

**Keywords:** albumin, ceftazidime, paracetamol, fluorescence, molecular dynamics, molecular docking

## Abstract

The coexistence of ceftazidime, which is a popular third-generation of cephalosporin antibiotic, with ubiquitous paracetamol or acetaminophen, is very likely because the latter is given to the patients to reduce fever due to bacterial infection along with an antibiotic such as the former. Therefore, in this study, we investigated the detailed binding of ceftazidime with plasma protein, human serum albumin (HSA), in the absence and presence of paracetamol using spectroscopic techniques such as fluorescence, UV-visible, and circular dichroism, along with in silico methods such as molecular docking, molecular dynamics simulations, and MM/PBSA-based binding free energy analysis. The basic idea of the interaction was attained by using UV-visible spectroscopy. Further, fluorescence spectroscopy revealed that there was a fair interaction between ceftazidime and HSA, and the mechanism of the quenching was a dynamic one, i.e., the quenching constant increased with increasing temperature. The interaction was, primarily, reinforced by hydrophobic forces, which resulted in the partial unfolding of the protein. Low concentrations of paracetamol were ineffective in affecting the binding of ceftazidime with has; although, a decrease in the quenching and binding constants was observed in the presence of high concentrations of the former. Competitive binding site experiments using warfarin and ibuprofen as site markers revealed that ceftazidime neither binds at drug site 1 or at drug site 2, articulating another binding site, which was confirmed by molecular docking simulations.

## 1. Introduction

Interaction of proteins with drugs is an important topic of study and draws the attention of researchers of many fields, such as medicine, biochemistry, pharmacy, chemistry, etc. [1]. In recent years, a lot of studies were performed on this topic and still numerous investigations are going on to understand the mechanisms of interactions of various substances with various types of proteins, enzymes, and receptors [1,2]. The importance of such interactions is due to the possible concurrences of these substances inside living beings. Moreover, several proteins also serve as the drug delivery agents and can be used in designing the drug delivery formulations [3,4,5,6].

Inside the human body, plasma proteins are the proteins that have a large probability of interactions with the drugs given to treat several illnesses [7]. There are several modes of administration of the drugs in which intravenous, intermuscular, and oral are amongst the most common modes. The drug injected through intravenous or intramuscular mode goes directly to the circulatory system, while the one given orally passes through the digestive system and is absorbed through small intensities then passed into the circulatory system [8,9]. It is the circulatory system that is responsible for the drugs being transported throughout the body, utilizing several carrier proteins such as serum albumin, globulin, and α-1-acid glycoprotein, so that it can reach the site of action [10].

Human serum albumin (HSA) is the most abundant protein in the blood that constitutes about 55% of the total plasma proteins and is responsible for controlling the blood osmotic pressure [11]. HSA is a large globular protein and is proficient in binding a large number of endogenous and exogeneous ligands due to its versatile structure, which has several binding sites that can accommodate a variety of ligands [12]. Due to the usefulness, binding efficiency, and prevalence, HSA is one of the most sought-after among scientists to understand its mechanisms with the drugs, small molecules, and ligands [13]. It is also a good candidate as a model protein for understanding protein–ligand interactions [14].

Ceftazidime (Drug Bank ID: DB00438) is a popular third-generation cephalosporin antibiotic used to treat bacterial infections. It is also given to the patients suffering from flu, cold, or viral infections. Ceftazidime is given to the patients suffering from fever due to infection that have a low white blood cell count. It can also be given in Whitmore’s disease that is an infection of the lungs and can range from mild bronchitis to severe pneumonia. It is also a beneficial drug in the case of certain wound infections and food poisoning. The anticancer activity of ceftazidime, in which it inhibits DLD-1 cells by inducing apoptotic cell death, was also reported very recently [15]. When ceftazidime given to the patients in the form of an injection given intravenously or intramuscularly, therefore, its interaction with HSA inside human body is highly anticipated. Thus, it would be interesting to study the interaction of ceftazidime with HSA. Recently, we studied the interaction of bovine serum albumin and lysozyme with ceftazidime in our previous works [16,17] and in the present study, we elaborated this work by seeing the effect of paracetamol on the binding.

It is renowned that paracetamol, also known as acetaminophen (Drug Bank ID: DB00316), is the most common medicines given to the patients suffering from fevers that happens due to the infection. Apart from this, it is also an effective drug to treat moderate pains. Paracetamol is commonly given orally in the form of tablets or syrups, which are absorbed by the small intestine through passive diffusion. It can also be given in the form of injection. Keeping in mind the possibility of co-existence of paracetamol and ceftazidime, particularly, in the bacterial or viral infections, we saw the effect of paracetamol on the binding of HSA and ceftazidime.

The use of computational methods in science and technology is increasing day by day. There are several approaches that are advantageous in drug designing and discovery. The combination of experimental and computational studies in a chemical or biochemical process gives a complete picture of its mechanism. Therefore, we applied both experimental and computation methods to study the interaction of ceftazidime with HSA in the absence and presence of paracetamol. The experimental studies were carried out using UV-visible, fluorescence, and circular dichroism spectroscopies, whereas in silico studies were accomplished using computational methods such as molecular docking, molecular dynamics simulations and MM/PBSA-based binding free energy analysis were performed.

## 2. Results and Discussions

### 2.1. UV-Visible Absorption Spectroscopy

**Figure 1:**
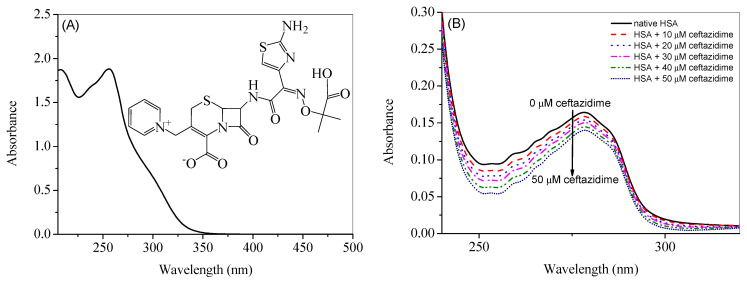
(**A**) UV-visible spectra of 100 µM ceftazidime at 25 °C. (B) Difference UV-visible spectra of HSA in the presence of various concentrations of ceftazidime.

The UV-visible spectrum of ceftazidime, along with its chemical structure, is given in Figure 1A. Ceftazidime shows a strong absorption peak at 255 nm and HSA a strong absorption at 280 nm, which is a prominent wavelength in the case of proteins containing aromatic amino acids such as tryptophan, tyrosine, and phenylalanine because of their absorption at this wavelength [18]. Therefore, the contribution of ceftazidime was subtracted from the UV-visible absorption spectra of HSA-ceftazidime complex (given in Figure 1B along with the UV-visible spectrum of pure HSA), which are considered as the difference spectra [19,20,21,22]. Figure 1B shows that the absorbance of HSA decreased constantly on the sequential addition of ceftazidime, which is most probably due to the interaction between the protein and drug.

### 2.2. Intrinsic Fluorescence

**Figure 2:**
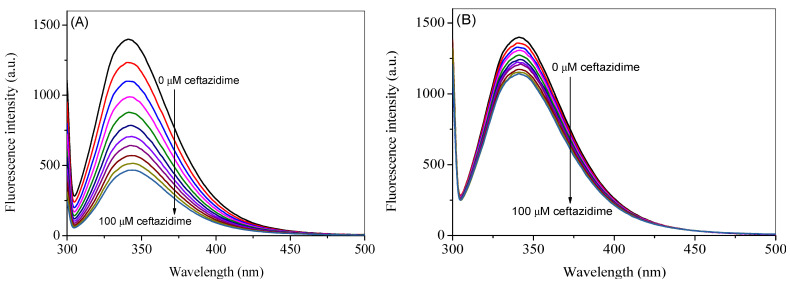
Observed (**A**) and corrected (**B**) fluorescence emission spectra of HSA in the presence of several concentrations of ceftazidime ranging from 0 to 100 µM with a constant increment of 10 µM at the excitation wavelength of 295 nm at 25 °C.

**Figure 3:**
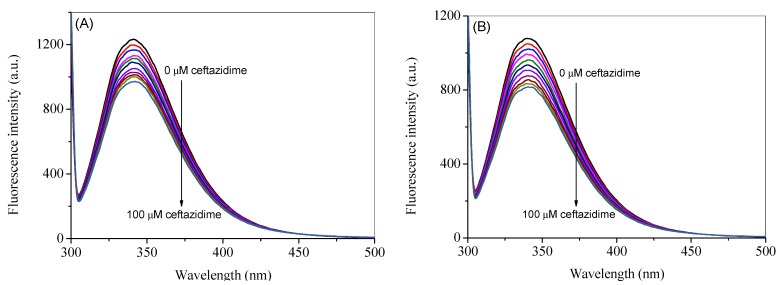
Corrected fluorescence emission spectra of HSA in the presence of several concentrations of ceftazidime ranging from 0 to 100 µM with a constant increment of 10 µM at the excitation wave-length of 295 nm at 35 °C (**A**) and 45 °C (**B**).

The intrinsic fluorescence of HSA is mainly contributed by a single tryptophan residue, which can be excited at 295 nm to give the maximum fluorescence emission at 340 nm [23]. The fluorescence emission spectra of HSA at the excitation wavelength of 295 nm in the absence and presence of ceftazidime at 25 °C are reported in Figure 2A. However, as can be seen from the UV-visible spectrum of pure ceftazidime, there is significant absorption at the selected excitation wavelength; therefore, the spectra were corrected for the inner filter effect using Equation (S1) and are presented in Figure 2B [20,21,24,25]. A noticeable change can be seen in corrected spectra as compared to the observed spectra. The observed spectra at higher temperatures (35 °C and 45 °C) are given in Figures S1 and S2, whereas the corresponding corrected spectra are shown in Figure 3A,B. It is evident from the figures that when ceftazidime is added to the HSA, it leads to a decrease in the fluorescence intensity, which is called fluorescence quenching due to the change in the microenvironment of the fluorophore(s). The corrected fluorescence data are used to further analyze the results given in the next section.

### 2.3. Analysis of Fluorescence Data

**Figure 4:**
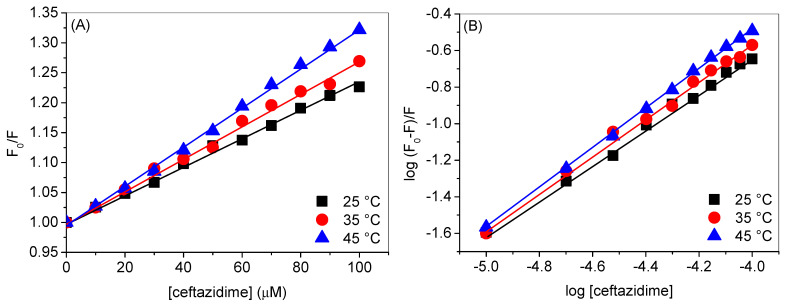
(**A**) Plots of *F*_0_/*F* vs. [ceftazidime] at various temperatures. (**B**) Plots of log (*F*_0_ − *F*)/*F* vs. log [ceftazidime] at various temperatures.

The fluorescence quenching is the result of either stable complex formation between fluorophore and the quencher or there is a collisional encounter between them. The first phenomenon is termed as static quenching, while the second one is known as dynamic quenching. The method to calculate the quenching constants was developed by Stern and Volmer [26], which is also called the Stern–Volmer equation and can be given as:

(1) $$\frac{F_{0}}{F}=1+K_{SV}[Q]$$

where *F*_0_ and *F* are the fluorescence intensities of HSA in the absence and presence of ceftazidime (quencher), [*Q*] is the concentration of quencher, and *K_SV_* is the Stern–Volmer quenching constant. The values of *K_SV_* (Table 1) can be obtained using Equation (1) from the plots of *F*_0_/*F* vs. [*Q*], which are given in Figure 4A. The involvement of the quenching mechanism, either static or dynamic, can be understood with the help of temperature variation. Dynamic quenching increases in increasing the temperature while static quenching constant decreases in such a case. The values of *K_SV_s* for HSA-ceftazidime interaction at various temperatures are given in Table 1. The *K_SV_* increased on increasing the temperature that indicates the involvement of the dynamic quenching mechanism. In our earlier work on ceftazidime–lysozyme interaction, the quenching mechanism was also a dynamic one.

The binding constant (*K_b_*) and number of binding sites (*n*) can also be estimated using the modified Stern–Volmer equation as [27]:

(2) $$log\frac{F_{0}-F}{F}=logK_{b}+nlog[Q].$$

*K_b_* and *n* were calculated using the plots of log(*F*_0_ − *F*)/*F* vs. log[ceftazidime] given in Figure 4B and their values are given in Table 1. The magnitude of the binding constant reveals that there was a good binding between HSA and ceftazidime and the binding ratio was approximately 1:1, i.e., the values of *n* were near to 1. The binding of ceftazidime with bovine serum albumin was found to be slightly stronger as compared to HSA, whereas lysozyme showed a weak binding with the former. Compared with the binding of HSA with other substances, such as indomethacin, safranal, and cuminaldehyde, these substances interacted more strongly with the former as compared to ceftazidime, whereas the binding of gemcitabine and cuminol was weaker [25,28,29,30]. Temperature rise resulted into the increase in the values of both *K_b_* as well as *n*. The increase in the *K_b_* with temperature is ascribed to the involvement of hydrophobic interactions in the binding, which were further evaluated using the widespread Van’t Hoff method in the next section.

### 2.4. Estimation of Thermodynamic Parameters

**Figure 5:**
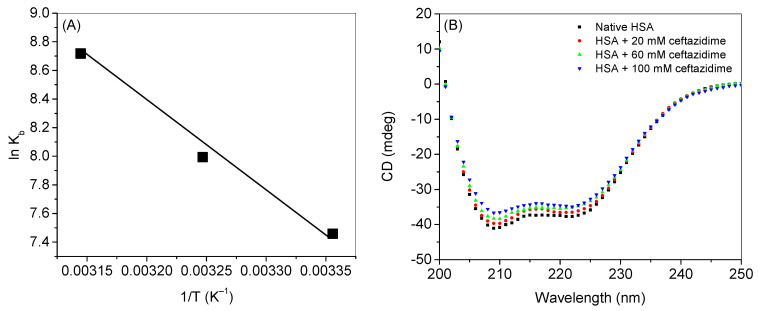
(**A**) Van‘t Hoff plots for the interaction of HSA with ceftazidime. (**B**) far-UV CD spectra of HSA in the absence and presence of several concentrations of ceftazidime.

Van’t Hoff equations (given in Equations (S2) and (S3)) can be used to evaluate the thermodynamic parameters such as the free energy change (Δ*G*), enthalpy change (Δ*H*), and entropy change (Δ*S*) of a process using the values of physical parameters at equilibrium. Herein, we used the binding constant, *K_b_*, at three different temperatures to fit into the Equation (S1) to obtain the values of Δ*H* from the slope and Δ*S* from the intercept of the plot of ln *K_b_* vs. 1/*T* (Figure 5A), which are given in Table 1. The values of Δ*H* and Δ*S* were, further, used to calculate Δ*G*. From the evaluation of thermodynamic parameters, involvement of the major forces in the binding can be understood along with the feasibility of the interaction. According a to report published by Ross and Subramanian [31], hydrogen bonding interactions dominate when the values of Δ*H* and Δ*S* are negative, whereas positive values of these parameters can be observed in case of hydrophobic interactions. In the present study, the negative values of Δ*G* indicated the feasibility of binding. Since the values of both Δ*H* and Δ*S* were positive, it can be deduced that the hydrophobic forces were the major forces involved in the binding.

### 2.5. Far-UV Circular Dichroism Spectroscopy

The change in the secondary structure of a protein can be monitored by studying the far-UV CD spectroscopy. HSA is an *α*-helical protein having around 67% *α*-helical contents, along with the small contributions of others [11]. Figure 5B displays the far-UV CD spectra of HSA in the absence and presence of ceftazidime. From the spectra, it is clear that ceftazidime affects the secondary structure if HSA and a partial unfolding of the protein take place, which is dependent on the concentration of the drug. This type of behavior of partial unfolding was also found in the case of ceftazidime interaction with bovine serum albumin and lysozyme, as well as in the case of cefoperazone binding with serum albumin [16,17,24].

### 2.6. Competitive Binding Site Assay

**Figure 6:**
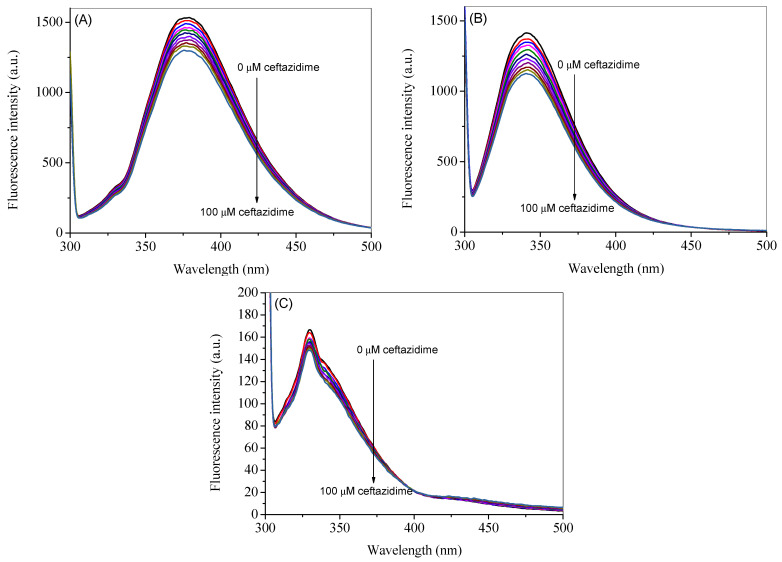
Corrected fluorescence spectra of HSA in the presence of several concentrations of ceftazidime ranging from 0 to 100 µM with a constant increment of 10 µM at the excitation wavelength of 295 nm at 25 °C in the presence of warfarin (**A**), ibuprofen (**B**), and hemin (**C**).

**Figure 7:**
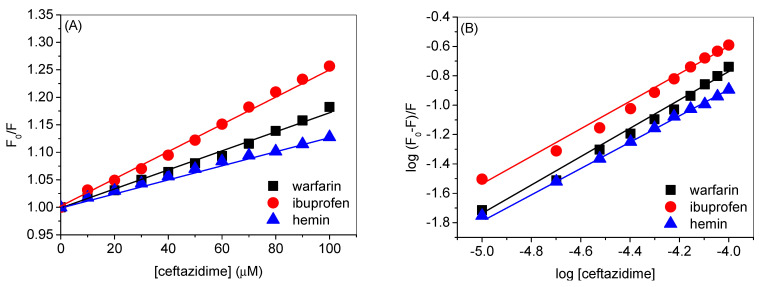
(**A**) Plots of *F*_0_/*F* vs. [ceftazidime] in presence of warfarin, ibuprofen, and hemin. (**B**) Plots of log (*F*_0_ − *F*)/*F* vs. log [ceftazidime] in the presence of warfarin, ibuprofen, and hemin.

HSA is a large globular protein with an atomic mass around 65 kDa, which is divided into three domains characterized as domain 1, domain 2, and domain 3, and each domain is, further, divided into two subdomains labeled as A and B. Due to its large structure, HSA has several binding sites, among which the two most common binding sites are the drug binding site 1 (DS1) or Sudlow’s site 1 and drug binding site 2 (DS2) or Sudlow’s site 2 [32,33]. DS1 is located in subdomain IIA, while DS2 is located in subdomain IIIA. There are several molecules that have the specific affinities towards these binding sites; for instance, warfarin binds strongly at DS1 and ibuprofen binds at DS2 [12]. There is another binding site within HSA, which is also known as fatty acid binding site 1, located in subdomain IB, which has a precise affinity for hemin [12]. Therefore, these molecules could be considered as the specific site markers for these sites and can be used to see the competitive binding between the molecule under consideration. The respective corrected fluorescence quenching spectra of HSA, complexed with warfarin, ibuprofen, and hemin, in the absence and presence of ceftazidime, are given in Figure 6A–C whereas the observed spectra are shown in the supporting info as Figures S3–S5. The corresponding quenching and binding plots are given in Figure 7A,B and the calculated values of quenching and binding constants are shown in Table 2. While ibuprofen does not affect both *K_SV_* as well as *K_b_*, these parameters decreased considerably in the presence of warfarin and even more with hemin. Thus, it can be understood that the binding site of ceftazidime within HSA is located either in subdomain IB or subdomain IIA, with a greater possibility within the former because the binding was least in that case. To obtain more understanding on the binding site, we performed computational studies using molecular docking and molecular dynamics simulations, which will be described in the next sub-sections.

### 2.7. Effect of Paracetamol on the Binding

**Figure 8:**
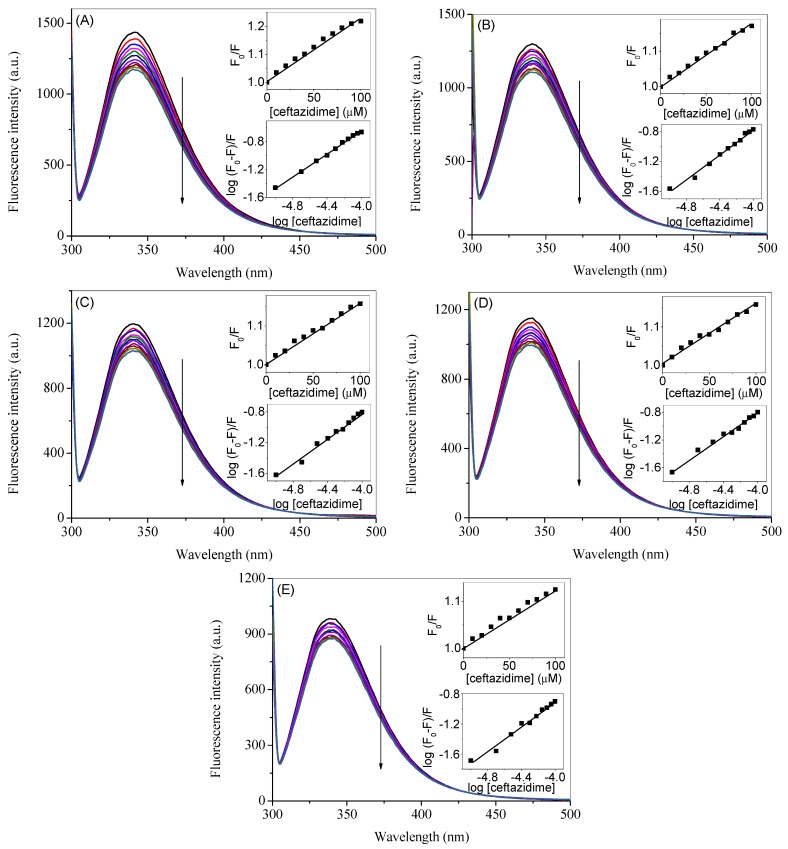
Corrected fluorescence spectra of HSA in the presence of several concentrations of ceftazidime ranging from 0 to 100 µM with a constant increment of 10 µM at the excitation wavelength of 295 nm at 25 °C in the presence of several concentrations of paracetamol 50 µM (**A**), 100 µM (**B**), 150 µM (**C**), 250 µM (**D**), and 500 µM (**E**). Inset 1 in each figure is the plots of *F*_0_/*F* vs. [ceftazidime] and inset 2 is the plots of log (*F*_0_ − *F*)/*F* vs. log [ceftazidime].

Due to the concurrence of both ceftazidime and paracetamol, as described in the introduction section, the effect of the latter was seen on the binding of the former with HSA. The corrected spectra of HSA–ceftazidime binding in presence of several concentrations of paracetamol, given in the parentheses, are shown in Figure 8A (50 µM), Figure 8B (100 µM), Figure 8C (150 µM), Figure 8D (250 µM), and Figure 8E (500 µM) whereas their corresponding observed spectra are given in Figures S6–S10. The respective quenching and binding plots are given in the insets of each of the figures and the values of *K_SV_* and *K_b_* of HSA–ceftazidime interaction in the presence of various paracetamol concentrations are enlisted in Table 3. A small concentration (50 µM) of paracetamol HSA has no effect on the binding; however, as its concentration increased further, the *K_SV_* and *K_b_* started to decrease. Which means that paracetamol could decrease the binding of HSA with ceftazidime.

**Figure 9:**
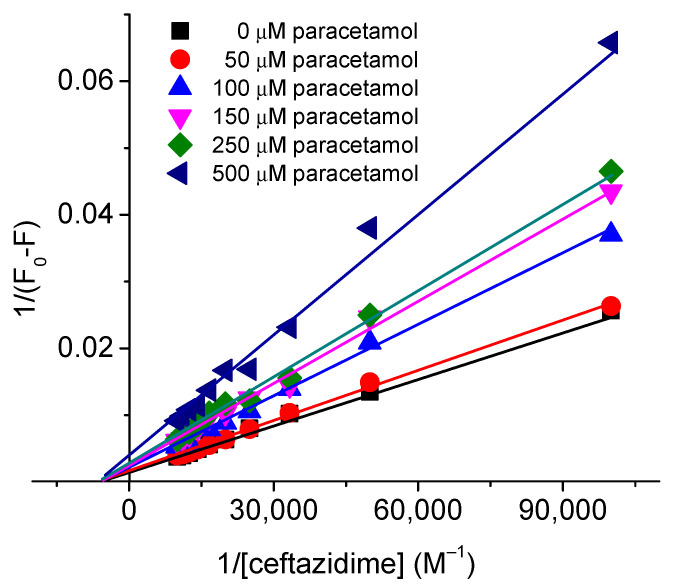
Lineweaver–Burk plots of paracetamol inhibition on HSA–ceftazidime binding.

To clarify the type of inhibition by paracetamol on the ceftazidime–HSA binding, the data were treated using the widespread Lineweaver–Burk equation [34]:

(3) $$\frac{1}{F_{0}-F}=\frac{1}{F_{0}K_{LB}\left[Q\right]}+\frac{1}{F_{0}}$$

where *K_LB_* is the Lineweaver–Burk constant that can be determined from the slope 1/(*F*_0_ × *K_LB_*) and intercept (1/*F*_0_) of the Lineweaver–Burk plots given in Figure 9.

The Lineweaver–Burk plots given in the Figure 9 show that the inhibition by paracetamol was the non-competitive type [35]. The values of *K_LB_*, given in Table S1 of the supporting info, decreased on increasing the concentration of paracetamol. It can be seen that the values of *K_LB_* are slightly higher than the one obtained from other method, but these are in the same order of magnitude and are in a comparable range. Further, it is obvious to obtain slightly different values of the parameters from different methods.

### 2.8. Molecular Docking

**Figure 10:**
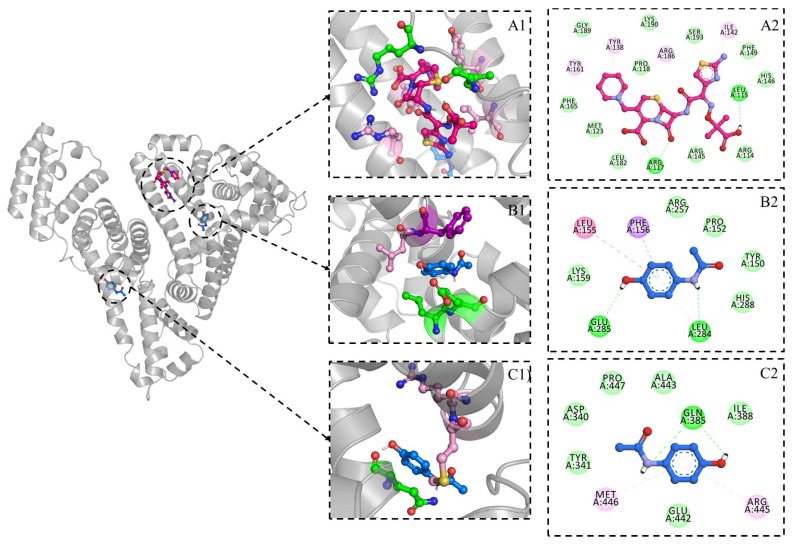
Docked orientation of ceftazidime with HSA (**A**), paracetamol in ceftazidime–HSA complex (**B**), and paracetamol with HSA (**C**). Figures with number 1 represent the docked orientation while figures numbered 2 are the 2D visualization of the intermolecular interactions observed in docking. The color scheme of interactions is as follows: green; hydrogen bond, pink; types of alkyl interactions, purple; pi–sigma interactions, and light green; Vander Waals interactions.

Ceftazidime in the molecular docking calculations displays a docked orientation in domain IB of HSA, which is in accordance with the competitive binding assay studies. The complex of HSA with ceftazidime shows an extensive network of interactions primarily via hydrophobic interactions, as observed via the fluorescence data, and shows a estimated binding affinity of −8.02 kcal/mol (estimated inhibition constant (*Ki*): 132.2 nM). Leu115 and Arg117 display the formation of hydrogen bonds while they also interact via alkyl interactions along with Arg186 and Ile142. Two pi–alkyl interactions are observed with Tyrosine residues, namely Tyr138 and Tyr161 (Figure 10 A). The influence of ceftazidime on the paracetamol–HSA complex is displayed via the site-directed docking of ceftizidime, which displays a docked orientation between the domain IB and IIA (estimated binding affinity: −9.62 kcal/mol; *Ki*: 88.78 nM). In this complex, paracetamol interacts via two hydrogen bonds with Leu284 and Glu285 while showing pi–alkyl interactions with Leu155 and a Pi–sigma interaction with Phe156 (Figure 10B). The blind docking of paracetamol displays a docked orientation in domain III, which is the probable preferred orientation of paracetamol with native HSA (estimated binding affinity: −5.23 kcal/mol; *Ki*: 146.7 µM). This complex displays interaction with Gln385 via hydrogen bond and Pi–alkyl interactions with Arg445 and Met446 (Figure 10C). The interaction analysis is summarized in Table 4.

### 2.9. Molecular Dynamics Simulations

**Figure 11:**
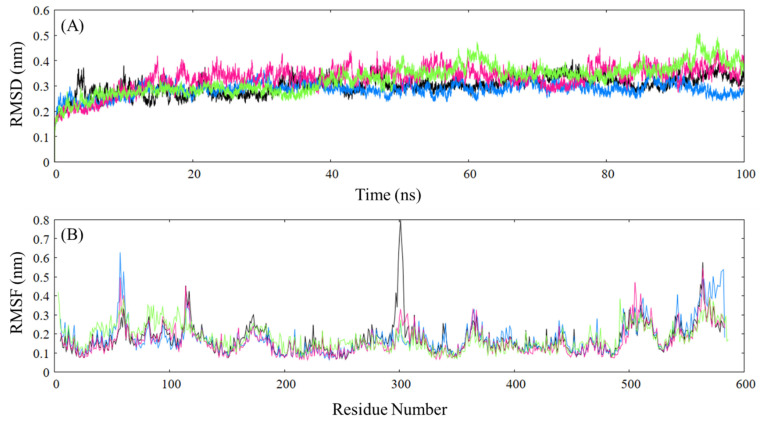
RMSD (**A**) and RMSF (**B**) plots for native HSA (black), paracetamol–HSA complex (blue), ceftazidime–HSA complex (hotpink), and paracetamol–ceftazidime–HSA (green).

The MD simulation results for HSA, along with its complexes with ceftazidime and paracetamol, were first analyzed with root mean square deviation (RMSD), which indicates the changes observed in the trajectory with respect to the initial structure. The free protein displays a variable trajectory in the first 50 ns, upon which it becomes considerably stable, yet a continuous trend is not obtained. The HSA–paracetamol complex shows a much more stable trajectory throughout the simulation, similar to the native protein with lower RMSD values (Figure 11A). The trajectory for HSA–ceftazidime shows an initial increase till 20 ns, upon which it shows a more stable trajectory with high and variable values for RMSD. Finally, the HSA complex with both paracetamol and ceftazidime initially remains similar to the paracetamol complex till 40 ns, upon which an increase in RMSD is observed, and the complex becomes more unstable. The analysis indicates a clear effect of paracetamol on ceftazidime’s interaction with HSA, as an increase in RMSD can be observed. Next, to understand the effect of the fluctuation at a residue level, root mean square fluctuation (RMSF) analysis is employed. RMSF analysis shows that the complexes mostly show similar patterns as that of the native, with notable changes occurring in domain I and domain IIIB of HSA (Figure 11B). The first observation can be made in the domain IA of HSA, where the binding of ligands induces significantly higher fluctuations (res 54–62). The second observation is made in the loop connecting domains IB and IIB, which shows an immensely high fluctuation in the native protein, yet upon ligand binding (both paracetamol and ceftazidime), a significant decrease can be observed in the loop. Since both fluctuations are observed in loop regions, they can be attributed as random motions.

**Figure 12:**
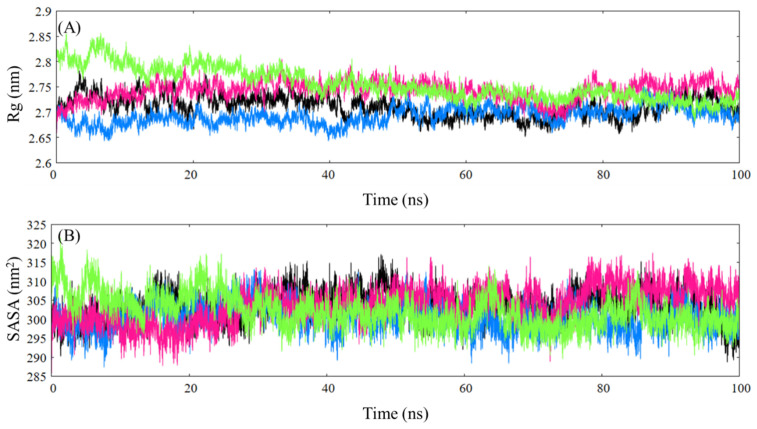
Rg (**A**) and SASA (**B**) plots for native HSA (black), paracetamol–HSA complex (blue), ceftazidime–HSA complex (hotpink), and paracetamol–ceftazidime–HSA (green).

Next, radius of gyration (Rg) and solvent-accessible surface area (SASA) analysis was performed to study the overall effect of paracetamol and ceftazidime on the overall fold, folding the properties and compactness of the protein. The Rg is a measure of the overall shape of the protein with respect to a central axis and provides information on the changes in the distribution of atoms in the protein along the axis. The native protein remains fairly stable in the Rg analysis, which indicates no significant changes in the protein fold; meanwhile, the paracetamol-HSA complex displays a decrease in Rg values in the initial 50 ns, upon which it displays a similar trajectory as the native protein, indicating a more compact structure (Figure 12A). The ceftazidime–HSA complex, on the other hand, displays slightly higher values of Rg, while the trajectory follows a similar pattern as the native protein, which shows an increase in protein looseness. Finally, the paracetamol–ceftazidime–HSA complex displays significantly higher Rg values at the beginning of the trajectory, which continues to decrease over the course of the simulation but remains higher than the native protein, which delineates a partial unfolding of the protein structure upon ceftazidime binding, which is not hindered upon paracetamol binding. These results are in congruence with the CD results presented in the earlier section. The SASA analysis describes the overall surface area of the protein that is in contact with the solvent environment, which delineates changes in the protein structure. The native protein displays a fairly linear trajectory with an average value of 303.34 nm^2^, which is similar to the ceftazidime–HSA complex (303.77 nm^2^) (Figure 12B). The paracetamol–HSA complex also displays a similar trajectory as the native protein, yet the SASA values are lower (300.57 nm^2^) than that of the native protein. An initial increase is seen in the paracetamol–ceftazidime–HSA complex, which continues to decrease (302.10 nm^2^), similar to that in Rg analysis. SASA analysis conveys that the changes in HSA structure upon ligand binding are not extremely apparent, yet only subtle changes occur in the protein.

**Figure 13:**
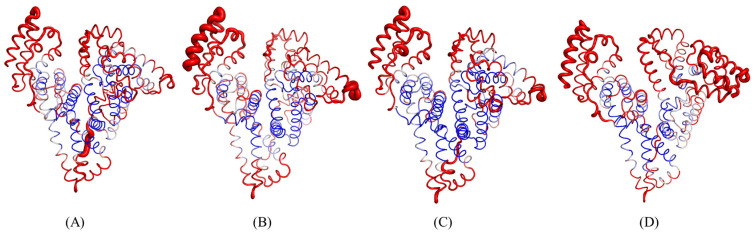
B-factor analysis for native HSA (**A**), paracetamol–HSA complex (**B**), ceftazidime–HSA complex (**C**), and paracetamol–ceftazidime–HSA (**D**). Red color indicates higher flexibility while blue represents the lowest. The thickness of the ribbon represents the extent of flexibility in the region.

The Define Secondary Structure of Proteins (DSSP) program allows us to analyze the changes induced in the secondary structure of the protein throughout the simulation. The results from DSSP show that the protein primarily consists of alpha helices (mainly alpha class) and loops, which remains consistent throughout the simulation for all complexes (Table 5). It is observed that no considerable changes occur in the secondary structure of HSA upon binding either of the ligands, which is in accordance with far-UV CD results. To better visualize the regions of flexibility in the structure of HSA, we additionally performed B-factors analysis on all systems where it can be observed that unbound HSA does not have much flexibility, which increases upon ligand binding (Figure 13). Most of the flexibility is limited to domains I and III of the protein. The interaction between the ligand molecules and protein receptors provides insights into the extent of binding, which is analyzed via hydrogen bonds observed throughout the simulation. Paracetamol, on average, maintains only one hydrogen bond throughout the simulation; meanwhile, ceftazidime maintains an average of two hydrogen bonds. In the paracetamol and ceftazidime complex with HSA, paracetamol does not form a considerable hydrogen bond on average, yet only shows dispersed bonds at different time points. On the contrary, ceftazidime shows a high number of hydrogen bonds in the first 15 ns, upon which they decrease while maintaining 2 hydrogen bonds on average during the simulation.

**Figure 14:**
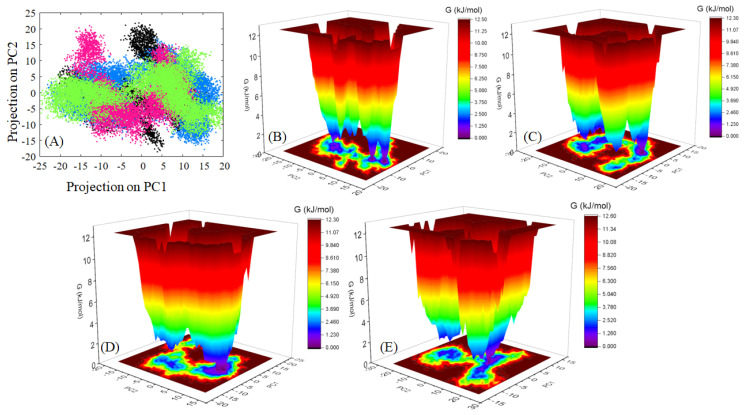
(**A**) PCA projections for native HSA (black), paracetamol–HSA complex (blue), ceftazidime–HSA complex (hot pink), and paracetamol–ceftazidime–HSA (green). FEL plots for native HSA (**B**), paracetamol–HSA complex (**C**), ceftazidime–HSA complex (**D**), and paracetamol–ceftazidime–HSA (**E**).

The principal component analysis of molecular dynamics trajectories applies the multivariate dimensionality reduction technique to identify essential or biologically relevant motions observed throughout the simulation based on the calculations of eigenvectors and their associated eigenvalues. These eigenvectors or principal components are subsequently projected onto an essential subspace where each point represents a distinct confirmation of the protein (Figure 14A). The dispersion of these states or confirmations on the essential subspace indicates the variance in different states, which can also be empirically studied by the trace of covariance matrix (tr(C)) value. The native protein displays a tr(C) value of 390.54 nm^2^ with dispersed confirmations on the essential subspace, which indicates a higher intrinsic mobility of HSA. The paracetamol–HSA complex also displays a dispersed cluster of confirmations with tr(C) values similar to that of the native protein, 390.521 nm^2^. The HSA complex with ceftazidime displays a decrease in the mobility of HSA, which is indicated by the tr(C) value of 338.86 nm^2^ and a more concerted distribution of states in the essential subspace. A similar result as the ceftazidime complex is observed for the paracetamol–ceftazidime HSA complex, where a slightly increased value of tr(C), 374.59 nm^2^, indicates that paracetamol decreases the stability of the complex and introduces perturbations in the confirmations. Next, PCA motions were visualized using the porcupine analysis, which displays motions as spikes. The directionality and the length of the spike represent the magnitude of the motion. The unbound HSA system shows PCA motions dispersed throughout the protein, while the ligand bound complexes display motions limited to domains I and III. The Paracetamol HSA complex displays dominant motions throughout the protein while the ceftazidime–HSA complex shows decreased motions throughout the protein. The paracetamol–ceftazidime-HSA complex shows a significant increase in PCA motions of domains I and III contributing to the instability in the complex (Figure S11). To understand the thermodynamic components of the confirmations observed in PCA, free energy landscape (FEL) plots were plotted and analyzed. The FEL plots define several energy basins, which are contoured as red, representing the highest energy states, with intermediate states as yellow followed by green, and ultimately, the lowest energy states are represented with blue followed by purple. In the FEL plot for native protein (Figure 14B), several energy minima can be observed, indicating the protein molecule’s inability to attain a low energy state; similar results are also observed for the paracetamol HSA complex (Figure 14C). The ceftazidime–HSA complex shows a concerted energy minimum in the plot, which indicates that upon ligand binding, the complex is able to attain a defined minimum energy state, indicating the stability of the confirmations in the minima (Figure 14D). Similarly, the paracetamol–ceftazidime–HSA complex is also able to attain a local minimum indicative of complex stability (Figure 14E).

**Figure 15:**
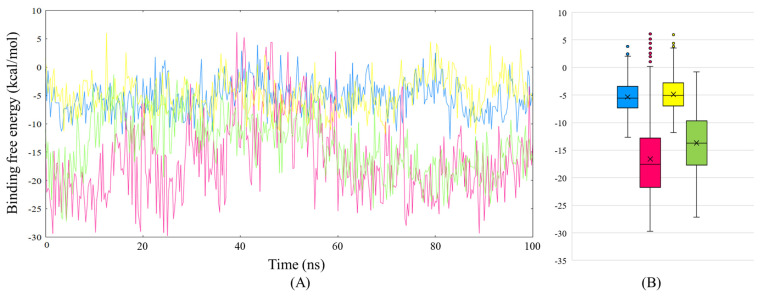
(**A**) MM/PBSA based binding free energy plot for paracetamol–HSA complex (blue), ceftazidime–HSA complex (hot pink), paracetamol in paracetamol–ceftazidime complex (yellow), and ceftazidime in paracetamol–ceftazidime complex (green). (**B**) Boxplots for MM/PBSA-based binding free energy analysis (color scheme is same as before).

Finally, the MM/PBSA-based binding free analysis was employed to understand better the ligands’ interaction with HSA, which provides the binding free energies for paracetamol and ceftazidime with HSA (Figure 15, Table 6). Ceftazidime displays an average binding free energy of −16.48 ± 6.95 kcal/mol, while the values vary significantly during the simulation, as displayed in Figure 15. During 35–60 ns of the simulation, ceftazidime shows poor binding affinity values going into positive values at some time points, which leads to an increase in the standard deviation observed in the average free energy value. This result can be attributed to the collisional quenching observed during fluorescence studies, which could indicate that while the interaction of ceftazidime with HSA is transient, the complex formed between the two is, however, stable as shown in the FEL plots. Next, paracetamol alone with HSA displays poor values for binding free energy, and the average value calculated is −5.34 ± 2.92 kcal/mol. Next, in the paracetamol–ceftazidime–HSA complex, paracetamol displays similar trends even at a different site, indicating the poor interaction ability of paracetamol with HSA. However, it is found that the interaction of paracetamol near the ceftazidime binding site causes a disruption between ceftazidime and HSA. A decrease in the binding free energy value is observed in ceftazidime (−13.68 ± 5.15 kcal/mol) when paracetamol is introduced to the complex, which is a result similar to that observed by the competitive binding site assay.

## 3. Materials and Methods

HSA essentially fatty acid free (≥99%, A3782), ceftazidime (≥90%, C3809), and paracetamol (BioXtra, ≥99.0%, A7085) were purchased from Sigma, USA. UV-visible studies were performed on a Perkin Elmer Lambda 45 spectrophotometer (Germany) within the range of 200 nm to 500 nm as required. Intrinsic fluorescence measurements were performed on a Hitachi F 7000 spectrofluorometer (Japan) equipped with the programmable temperature controller. The excitation and emission slit widths were adjusted to 5 nm with a PMT voltage of 500 V. Circular dichroism (CD) spectrophotometric recordings were obtained with a Jasco J-815 spectropolarimeter (Japan) using a quartz cuvette of 0.1 nm. The studies were carried out in the 20 mM tris buffer of pH 7.4 and at 25 °C unless stated otherwise. Autodock 4.2 [36] was used to perform molecular docking and MD simulations were performed through GROMACS 2021.3 [37] with the utilization of the CHARMM force field for a production run of 100 ns. The detailed methodology is given in the Supplementary Materials.

## 4. Conclusions

The detailed study of the interaction of human serum albumin (HSA) with a popular antibiotic drug, ceftazidime, was performed using several spectroscopic techniques along with in silico approaches. Because of the very large probability of coexistence of ceftazidime with a popular nonsteroidal anti-inflammatory drug, paracetamol, the effect of the latter was also seen on the interaction. Ceftazidime interacted with HSA through a dynamic quenching mechanism with approximately a 1:1 binding ratio. The preferred binding site of ceftazidime within HSA was located in subdomain IB and the interaction led to the partial unfolding of HSA. Small concentrations of paracetamol did not influence the binding of HSA with ceftazidime, although, higher concentrations decreased the binding significantly. The major forces involved in the binding were hydrophobic forces.

## Figures and Tables

**Table 1:** Stern–Volmer constants, binding constants, and thermodynamic parameters of the interaction of HSA with ceftazidime at various temperatures.

Temperature (°C)	Quenching Parameters			Binding Parameters		Thermodynamic Parameters		
	*K_SV_* (M^–1^)	R^2^	*n*	*K_b_* (M^–1^)	R^2^	Δ*G* (kJ mol^−1^)	Δ*H* (kJ mol^−1^)	Δ*S* (J mol^−1^ K^−1^)
25	2.3 × 10^3^	0.9957	0.96	1.7 × 10^3^	0.9966	−18.4	49.5	227.8
35	2.6 × 10^3^	0.9942	1.01	3.0 × 10^3^	0.9944	−20.7		
45	3.2 × 10^3^	0.9967	1.09	6.1 × 10^3^	0.9992	−23.0		

**Table 2:** Stern–Volmer constants and binding constants of the interaction of HSA with ceftazidime in the presence of warfarin, ibuprofen, and hemin as site markers.

Site Marker	*K_SV_* (M^–1^)	*K_b_* (M^–1^)
Warfarin	1.7 × 10^3^	1.5 × 10^3^
Ibuprofen	2.3 × 10^3^	2.6 × 10^3^
Hemin	1.3 × 10^3^	0.4 × 10^3^

**Table 3:** Stern–Volmer constants and binding constants of the interaction of HSA with ceftazidime in the presence of several concentrations of paracetamol.

[Paracetamol] (µM)	*K_SV_* (M^–1^)	*K_b_* (M^–1^)
50	2.2 × 10^3^	1.4 × 10^3^
100	1.8 × 10^3^	0.45 × 10^3^
150	1.7 × 10^3^	0.40 × 10^3^
250	1.6 × 10^3^	0.30 × 10^3^
500	1.3 × 10^3^	0.25 × 10^3^

**Table 4:** Molecular docking interaction analysis of HSA complexes.

S\No.	Ligands ID	Binding Affinity (kcal/mol)	HB	D (Å)	Pi-SR	D (Å)	vdWISR
1	Ceftazidime	−8.02	Leu115 Arg117	2.25 2.71 2.94	Leu115 Arg117 Tyr138 Ile142 Tyr161 Tyr138	4.62 4.84 4.47 4.33 5.08 4.13 4.33	Arg114, Pro118, Met123, Arg145, His146, Phe149, Phe165, Leu182, Gly189, Lys190, Ser193
2	Paracetamol	−5.23	Leu284 Glu285	2.35 2.32	Leu155 Phe156 Leu284	5.01 3.75 4.65	Tyr150, Pro152, Lys159, Arg257, His288
3	Ceftazidime with paracetamol-HSA complex	−9.62	Gln385	2.22 2.25	Arg445 Met446	4.84 5.46	Asp340, Tyr341, Ile388, Glu442, Ala443, Pro447

**Table 5:** Percentages of secondary structural elements of HSA and its complexes.

System	Structure	Coil	Bend	Turn	α-Helix	5-Helix	3-Helix
HSA	78	14	7	8	70	0	1
Paracetamol–HSA	78	13	6	8	71	0	2
Ceftazidime–HSA	78	14	7	8	69	0	1
Paracetamol–ceftazidime–HSA	78	13	7	8	70	0	2

**Table 6:** MM/PBSA-based binding free energy values for HSA and its complexes.

System	Van der Waals Energy (kcal/mol)	Electrostatic Energy (kcal/mol)	Polar Solvation Energy (kcal/mol)	SASA Energy (kcal/mol)	Binding Free Energy (kcal/mol)
HSA–Paracetamol	−18.97 ± 2.00	−12.61 ± 2.85	28.66 ± 5.00	−2.41 ± 0.17	−5.34 ± 2.92
HSA–ceftazidime	−38.49 ± 8.07	−38.18 ± 14.99	65.13 ± 18.46	−5.06 ± 0.55	−16.48 ± 6.95
HSA–paracetamol–ceftazidime					
(i) Paracetamol	−19.27 ± 1.99	−4.62 ± 2.31	−4.62 ± 2.31	2.69 ± 0.16	−4.87 ± 2.82
(ii) Ceftazidime	−43.08 ± 4.81	−31.04 ± 11.58	66.22 ± 14.21	−5.71 ± 0.33	−13.68 ± 5.15

## Data Availability

All data have been included in the manuscript.

## Supplementary Materials

The supporting information can be downloaded at: https://www.mdpi.com/article/10.3390/ijms241914811/s1. References [38,39] are cited in the supplementary materials.

## References

[1] Ballante F. (2021). Protein-Ligand Interactions and Drug Design.

[2] Vuignier K., Schappler J., Veuthey J.-L., Carrupt P.-A., Martel S. (2010). Drug–protein binding: A critical review of analytical tools. Anal. Bioanal. Chem..

[3] Molino N.M., Wang S.-W. (2014). Caged protein nanoparticles for drug delivery. Curr. Opin. Biotech..

[4] Bidwell G., Reese C., Shao Q.M., Chade A. (2015). A Kidney-targeted Protein Biopolymer Drug Delivery System. Faseb. J..

[5] Kianfar E. (2021). Protein nanoparticles in drug delivery: Animal protein, plant proteins and protein cages, albumin nanoparticles. J. Nanobiotechnol..

[6] Hong S., Choi D.W., Kim H.N., Park C.G., Lee W., Park H.H. (2020). Protein-Based Nanoparticles as Drug Delivery Systems. Pharmaceutics.

[7] Scheife R.T. (1989). Protein Binding: What Does it Mean?. DICP.

[8] Alagga A.A., Gupta V. (2023). Drug Absorption. StatPearls.

[9] Aldred E.M., Buck C., Vall K., Aldred E.M., Buck C., Vall K. (2009). Chapter 16—How do drugs get into cells. Pharmacology.

[10] Simmons P., Penny R., Goller I. (1969). Plasma Proteins a Review. Med. J. Aust..

[11] Theodore Peters J. (1995). All About Albumin: Biochemistry, Genetics, and Medical Applications.

[12] Ghuman J., Zunszain P.A., Petitpas I., Bhattacharya A.A., Otagiri M., Curry S. (2005). Structural basis of the drug-binding specificity of human serum albumin. J. Mol. Biol..

[13] Fanali G., di Masi A., Trezza V., Marino M., Fasano M., Ascenzi P. (2012). Human serum albumin: From bench to bedside. Mol. Asp. Med..

[14] Zvetanka D.Z. (2015). Studies on Drug—Human Serum Albumin Binding: The Current State of the Matter. Curr. Pharm. Des..

[15] Pfab C., Abgaryan A., Danzer B., Mourtada F., Ali W., Gessner A., El-Najjar N. (2022). Ceftazidime and cefepime antagonize 5-fluorouracil’s effect in colon cancer cells. BMC Cancer.

[16] Ali M.S., Waseem M., Subbarao N., Al-Lohedan H.A. (2021). Dynamic interaction between lysozyme and ceftazidime: Experimental and molecular simulation approaches. J. Mol. Liq..

[17] Ali M.S., Muthukumaran J., Al-Lohedan H.A. (2020). Molecular interactions of ceftazidime with bovine serum albumin: Spectroscopic, molecular docking, and DFT analyses. J. Mol. Liq..

[18] Schmid F.X. (2001). Biological Macromolecules: UV-visible Spectrophotometry. eLS.

[19] Ali M.S., Al-Lohedan H.A. (2017). Deciphering the interaction of procaine with bovine serum albumin and elucidation of binding site: A multi spectroscopic and molecular docking study. J. Mol. Liq..

[20] Ali M.S., Muthukumaran J., Jain M., Al-Lohedan H.A., Abul Farah M., Alsowilem O.I. (2021). Experimental and computational investigation on the binding of anticancer drug gemcitabine with bovine serum albumin. J. Biomol. Struct. Dyn..

[21] Ali M.S., Muthukumaran J., Jain M., Al-Sanea A.S.S., Al-Lohedan H.A. (2021). Experimental and in silico investigation on the interaction of indomethacin with bovine serum albumin: Effect of sodium dodecyl sulfate surfactant monomers on the binding. J. Mol. Liq..

[22] Ali M.S., Rehman M.T., Al-Lohedan H.A., AlAjmi M.F. (2022). Exploration of the binding between cuminol and bovine serum albumin through spectroscopic, molecular docking and molecular dynamics methods. J. Biomol. Struct. Dyn..

[23] Lakowicz J.R. (2006). Principles of Fluorescence Spectroscopy.

[24] Ali M.S., Muthukumaran J., Jain M., Santos-Silva T., Al-Lohedan H.A., Al-Shuail N.S. (2021). Molecular interactions of cefoperazone with bovine serum albumin: Extensive experimental and computational investigations. J. Mol. Liq..

[25] Ali M.S., Al-Lohedan H.A. (2018). Spectroscopic and computational evaluation on the binding of safranal with human serum albumin: Role of inner filter effect in fluorescence spectral correction. Spectrochim. Acta A.

[26] Stern O., Volmer M. (1919). The extinction period of fluorescence. Phys. Z.

[27] Jiang M., Xie M.X., Zheng D., Liu Y., Li X.Y., Chen X. (2004). Spectroscopic studies on the interaction of cinnamic acid and its hydroxyl derivatives with human serum albumin. J. Mol. Struct..

[28] Ali M.S., Muthukumaran J., Jain M., Tariq M., Al-Lohedan H.A., Al-Sanea A.S.S. (2023). Detailed Experimental and In Silico Investigation of Indomethacin Binding with Human Serum Albumin Considering Primary and Secondary Binding Sites. Molecules.

[29] Ali M.S., Al-Lohedan H.A. (2022). Experimental and Computational Investigation on the Interaction of Anticancer Drug Gemcitabine with Human Plasma Protein: Effect of Copresence of Ibuprofen on the Binding. Molecules.

[30] Ali M.S., Rehman M.T., Al-Lohedan H., AlAjmi M.F. (2022). Spectroscopic and Molecular Docking Investigation on the Interaction of Cumin Components with Plasma Protein: Assessment of the Comparative Interactions of Aldehyde and Alcohol with Human Serum Albumin. Int. J. Mol. Sci..

[31] Ross P.D., Subramanian S. (1981). Thermodynamics of protein association reactions: Forces contributing to stability. Biochemistry.

[32] Sudlow G., Birkett D.J., Wade D.N. (1975). The characterization of two specific drug binding sites on human serum albumin. Mol. Pharmacol..

[33] Sudlow G., Birkett D.J., Wade D.N. (1976). Further Characterization of Specific Drug Binding-Sites on Human-Serum Albumin. Mol. Pharmacol..

[34] Zhang H.X., Huang X., Mei P., Li K.H., Yan C.N. (2006). Studies on the interaction of tricyclazole with beta-cyclodextrin and human serum albumin by spectroscopy. J. Fluoresc..

[35] Enna S.J., David B. (2007). Robert Roskoski, in Modulation of Enzyme Activity, Bylund, xPharm: The Comprehensive Pharmacology Reference.

[36] Morris G.M., Huey R., Lindstrom W., Sanner M.F., Belew R.K., Goodsell D.S., Olson A.J. (2009). AutoDock4 and AutoDockTools4: Automated docking with selective receptor flexibility. J. Comput. Chem..

[37] Van Der Spoel D., Lindahl E., Hess B., Groenhof G., Mark A.E., Berendsen H.J.C. (2005). GROMACS: Fast, flexible, and free. J. Comput. Chem..

[38] Zoete V., Cuendet M.A., Grosdidier A., Michielin O. (2011). SwissParam: A fast force field generation tool for small organic molecules. J. Comput. Chem..

[39] Kumari R., Kumar R., Lynn A. (2017). g_mmpbsa—A GROMACS Tool for High-Throughput MM-PBSA Calculations. J. Chem. Inf. Model..

