# Supplementary material for: Non-Steroidal Anti-Inflammatory Drug Effect on the Binding of Plasma Protein with Antibiotic Drug Ceftazidime: Spectroscopic and In Silico Investigation
Source: Int J Mol Sci. 2023 Sep 30;24(19):14811. doi: 10.3390/ijms241914811 (PMC10573175; doi:10.3390/ijms241914811)
Supplement: Supplementary file 1 [file ijms-24-14811-s001.zip › ijms-2613967-supplementary.pdf]

Non-steroidal anti-inflammatory drug effect on the binding of plasma protein with antibiotic drug ceftazidime: Spectroscopic and in silico investigation

Supporting Information

UV–Visible spectra were recorded between the wavelength range of 200 to 400 nm on Perkin-Elmer Lambda 45 Spectrophotometer equipped with autosampler and water-bath with temperature controller. Quartz cuvettes of 1 cm path length were used for the measurements.

Fluorescence measurements were performed on Hitachi spectrofluorometer (Model F 7000) equipped with a PC and programmable temperature controller. Unless stated, the fluorescence spectra were collected at 25 °C with a cell of path length 1 cm. The excitation and emission slits were set at 5 nm. Intrinsic fluorescence was measured by exciting lysozyme at 280 nm and 295 nm.

The circular dichroism studies of lysozyme in presence of drug (ceftazidime) were carried out with JASCO J-815 spectropolarimeter equipped with a Peltier-type temperature controller. The instrument was calibrated with d-10-camphorsulfonic acid. All the CD spectra were collected in a cell of 0.1 mm path-length. The scan speed was 100 nm/min and response time of 1 s for all measurements. Each spectrum was the average of 3 scans.

The structure for HSA was obtained from Research Collaboratory Structural Bioinformatics – Protein Data Bank (RCSB-PDB) from ID "1AO6" (https://www.rcsb.org/structure/1ao6) which was then prepared for molecular docking studies by removing water molecules, any ligands and subsequently all non-polar hydrogens were merged, polar hydrogens were added along with kollman charges via MGLTools 1.5.7 as recorded as PDBQT (XYZ coordinates + Partial charges + Atom type)

format. The 3D coordinates for Paracetamol (DrugBank ID: DB00316; URL: https://go.drugbank.com/drugs/DB00316) and Ceftazidime (DrugBank ID: DB00438; URL: https://go.drugbank.com/drugs/DB00438) were retrieved from DrugBank database and prepared similarly as HSA with the exception of adding gasteiger charges instead of kollman charges. Initially both the ligands were blind docked to the entire HSA molecule with gridbox dimensions (npts) as x_center = 21.135, y_center = 32.955, z_center = 23.206 with grid points 126 for all coordinates and a grid spacing value of 0.375. Autodock v4.2 [1]was run with Lamarckian Genetic Algorithm (LGA) program with a run size of 2000, population size of 300, maximum number of generations was 27,000 and maximum number of evaluations was 25,00,000. The clusters obtained from the Autodock results were analysed and suitable complexes were extracted. The HSA-paracetamol complex was further docked with ceftazidime in a site specific manner (x_grid points = 70, y_grid points = 82, z_grid points = 76 and x_center = 33.56, y_center = 32.495, z_center = 37.064) with similar parameters as described earlier to understand the influence of paracetamol on the binding of ceftazidime. The complexes obtained from Autodock were analyzed via Biovia Discovery Studio for intermolecular interactions and were subsequently utilized for molecular dynamics simulations.

Molecular Dynamics (MD) simulations were performed through GROMACS 2021.3 [2] with the utilization of CHARMM force field for a production run of 100 ns. The topology for HSA was generated through the *gmx pdb2gmx* module while the topology for the ligands were prepared via SWISSPARAM server using mol2 as input format with all hydrogens [3]. The protein-ligand complex was defined to a unit cell with a -d value of 1.0 and -bt category was assigned as cubic. The unit cell was then solvated with TIP4P

water model along with charge neutralization via the addition of Na ions. The solvated system was then minimized using the steepest descent algorithm to a tolerance value of 100 kJ/mol/nm. The minimized systems were further subjected to temperature and pressure equilibration steps at 300 K and 1 bar, respectively. Finally, the production MD was performed for a time of 100 ns which included 50 million steps at a time interval of 2 fs. The Molecular Mechanics Poisson-Boltzmann Surface area, hereafter written as MM/PBSA (binding free energy analysis), was performed using the MMPBSA as defined by Kumari et al [4] to the entire MD run at a time interval of 100 ps. The results from the MD production run were analyzed via various GROMACS in-built commands and plotted through Gnuplot. The initial uncorrected trajectories obtained from the MD simulations, both for unbound and ligand-bound HSA complexes, were processed to remove periodic boundary conditions (PBC) using the *gmx trjconv* command. These corrected trajectories were subsequently used for a range of structural analyses utilizing various GROMACS commands, including *gmx rms* (Root Mean Square Deviation), *gmx rmsf* (Root Mean Square Fluctuation), *gmx gyrate* (Radius of Gyration), *gmx sasa* (Solvent Accessible Surface Area), *gmx hbonds* (Intermolecular hydrogen bonds), *gmx do_dssp* (secondary structural analysis), *gmx covar, gmx anaeig* (Principal Component Analysis for essential dynamics), and *gmx sham* (free energy landscape).

Equation used for inner filter effect correction:

The observed fluorescence spectra were corrected with following equation:

$$F_{corr} = F_{obs} \times 10^{(A_{exi} + A_{emi})/2} \quad \text{(S1)}$$

where, F_{corr} and F_{obs} are the corrected and observed fluorescence emission intensities, of lysozyme respectively, A_{exi} and A_{emi} are the absorbances of drug at the excitation and emission wavelengths, respectively.

Equation used to calculate thermodynamic parameters:

van 't Hoff equation is given as:

$$\ln K_b = \frac{-\Delta H}{RT} + \frac{\Delta S}{R} \qquad \text{(S2)}$$

$$\Delta G = \Delta H - T\Delta S \qquad \text{(S3)}$$

where ΔH is enthalpy change, ΔS is entropy change and ΔG is free energy change. R is gas constant and T is temperature in K.

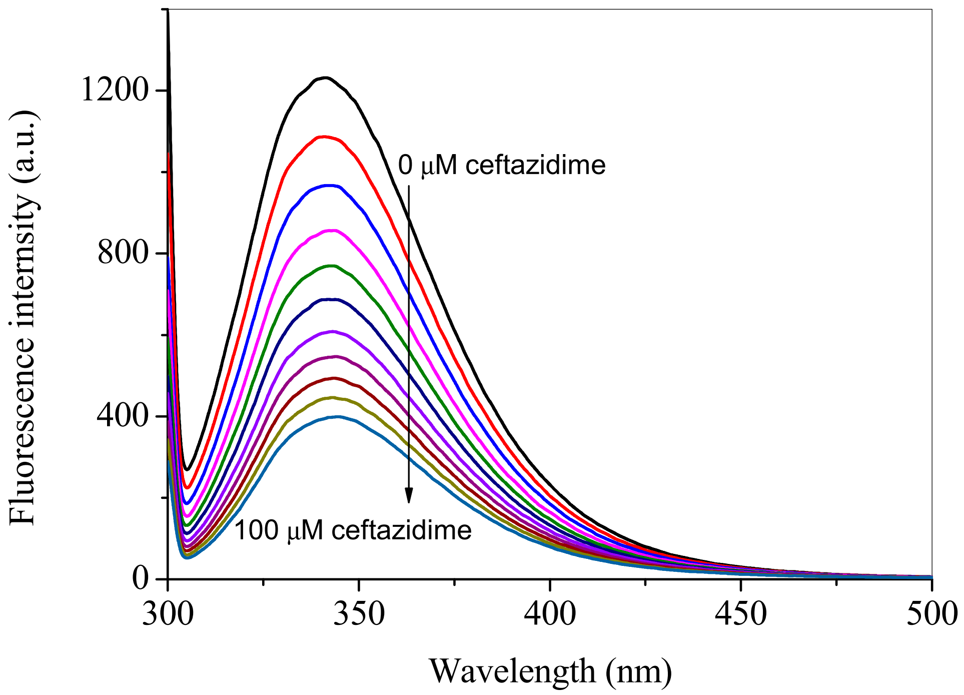

Figure S1. Observed fluorescence emission spectra of HSA in presence of several concentrations of ceftazidime ranging from 0 to 100 μM with a constant increment of 10 μM at the excitation wavelength of 295 nm at 35 °C.

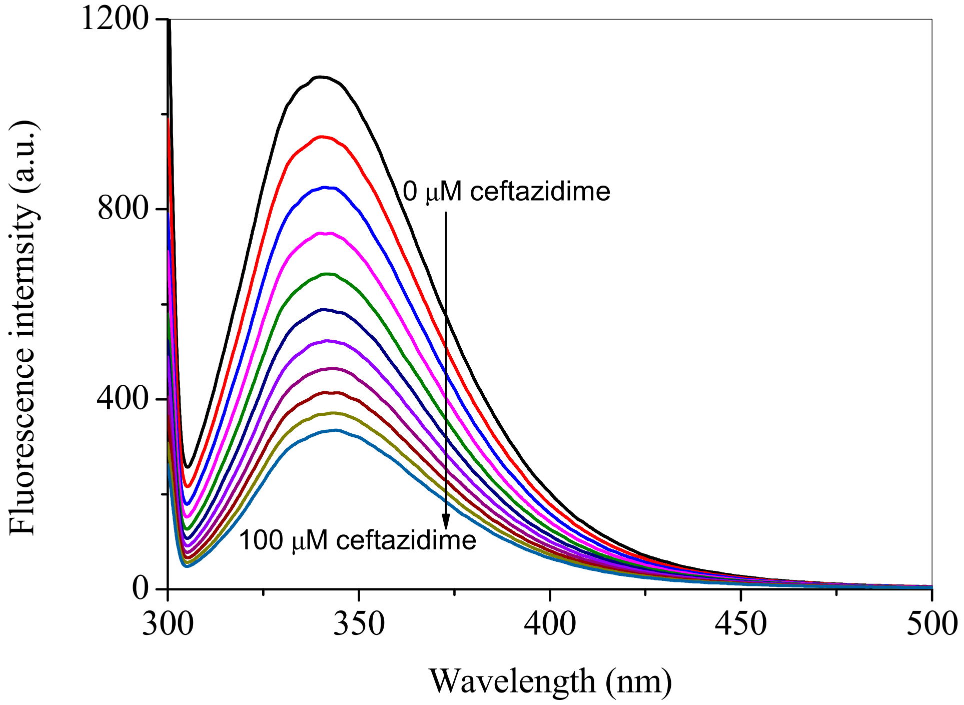

Figure S2. Observed fluorescence emission spectra of HSA in presence of several concentrations of ceftazidime ranging from 0 to 100 μM with a constant increment of 10 μM at the excitation wavelength of 295 nm at 45 °C.

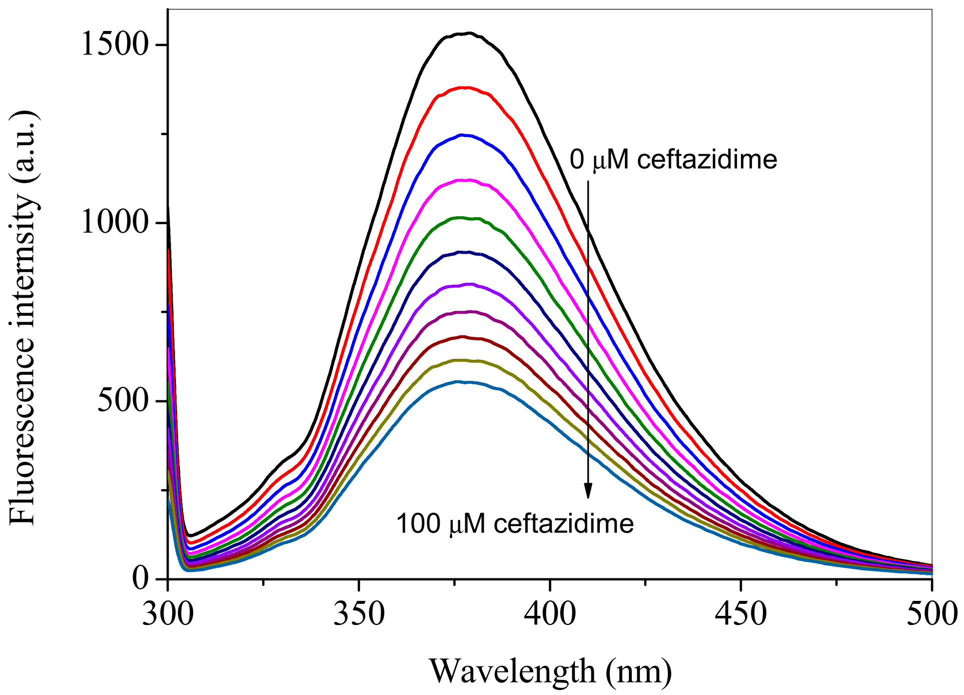

Figure S3. Observed fluorescence spectra of HSA in presence of several concentrations of ceftazidime ranging from 0 to 100 μM with a constant increment of 10 μM at the excitation wavelength of 295 nm at 25 °C in presence of warfarin.

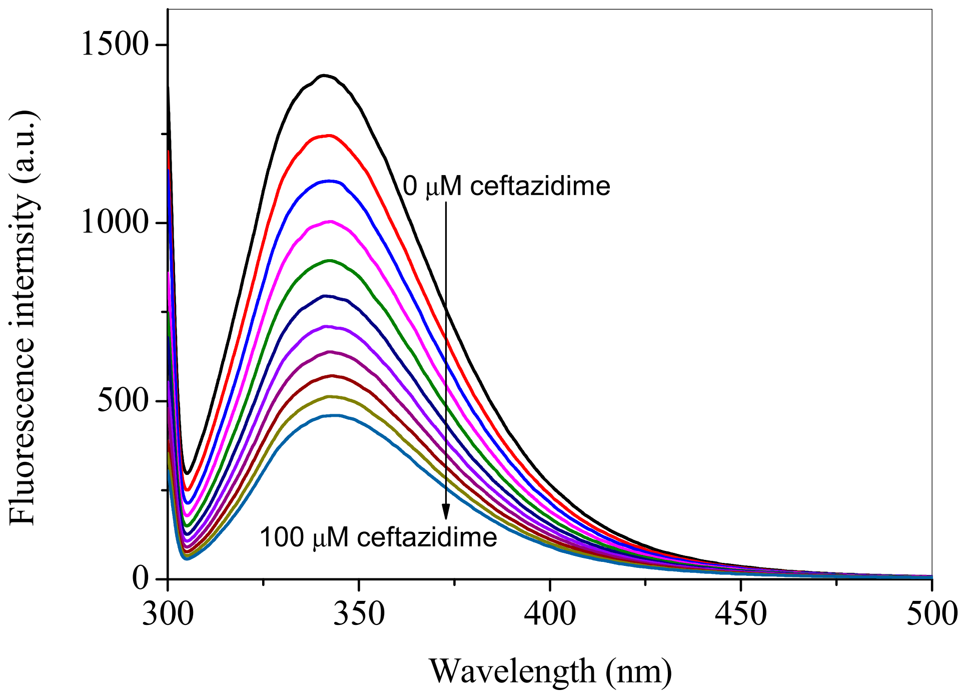

Figure S4. Observed fluorescence spectra of HSA in presence of several concentrations of ceftazidime ranging from 0 to 100 μM with a constant increment of 10 μM at the excitation wavelength of 295 nm at 25 °C in presence of ibuprofen.

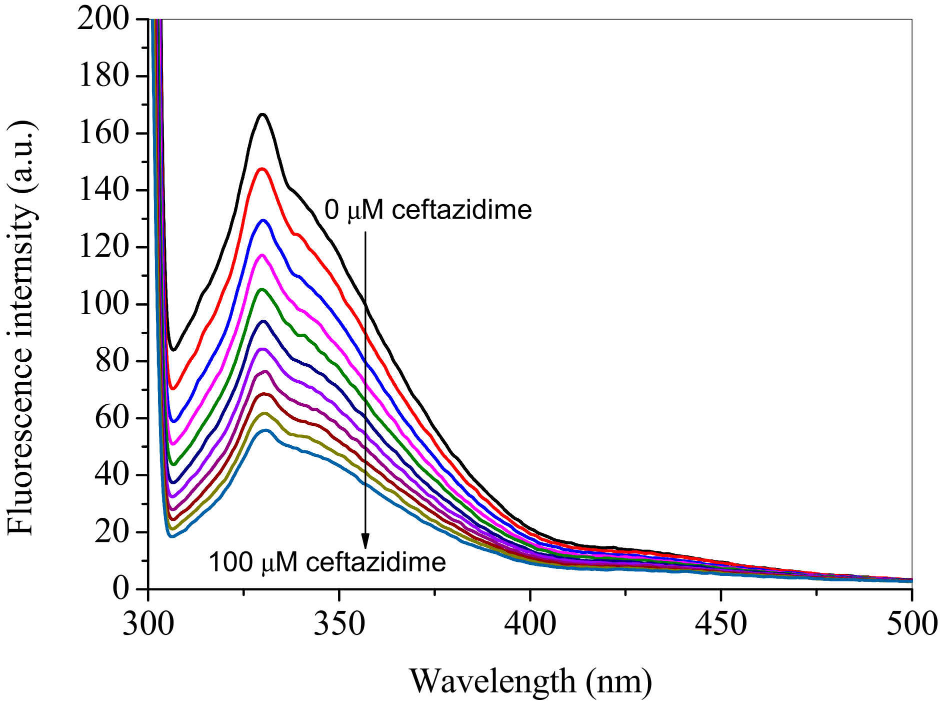

Figure S5. Observed fluorescence spectra of HSA in presence of several concentrations of ceftazidime ranging from 0 to 100 μM with a constant increment of 10 μM at the excitation wavelength of 295 nm at 25 °C in presence of hemin.

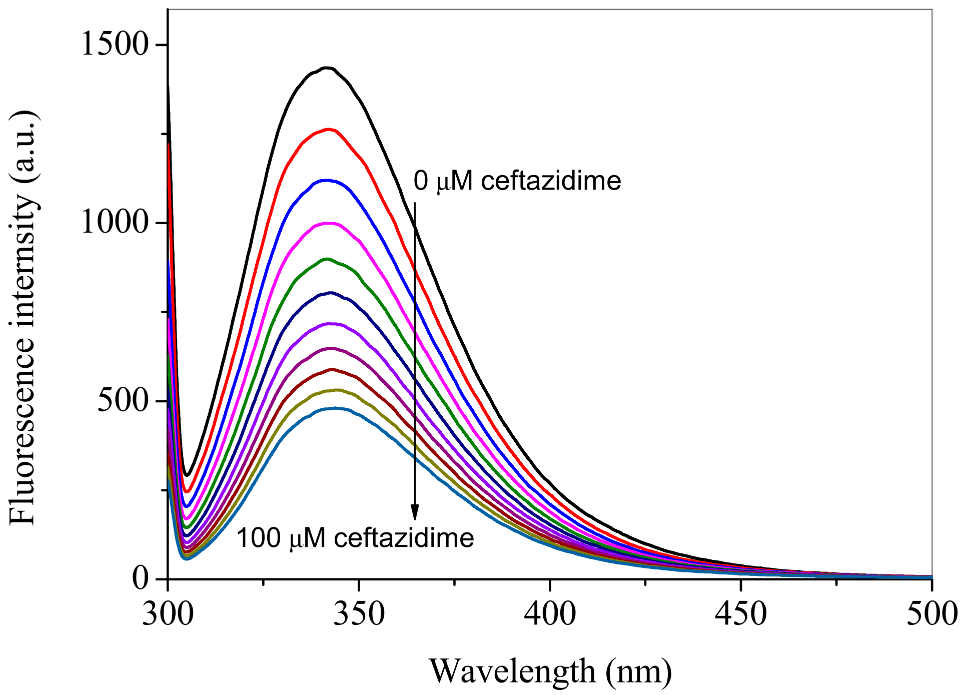

Figure S6. Observed fluorescence spectra of HSA in presence of several concentrations of ceftazidime ranging from 0 to 100 μM with a constant increment of 10 μM at the excitation wavelength of 295 nm at 25 °C in presence of several concentrations of 50 μM paracetamol.

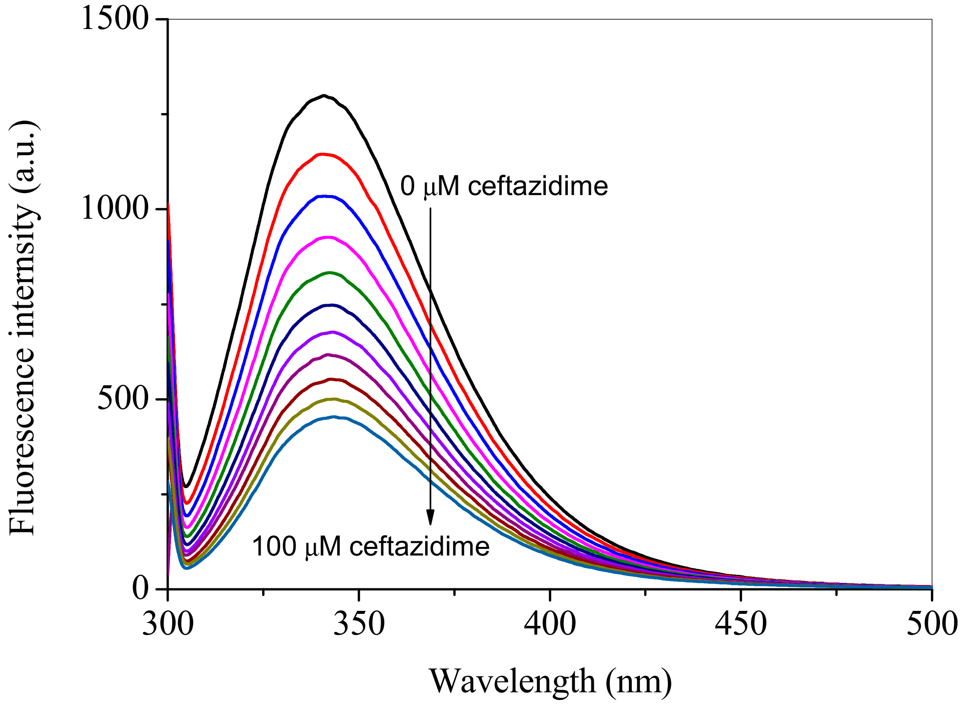

Figure S7. Observed fluorescence spectra of HSA in presence of several concentrations of ceftazidime ranging from 0 to 100 μM with a constant increment of 10 μM at the excitation wavelength of 295 nm at 25 °C in presence of several concentrations of 100 μM paracetamol.

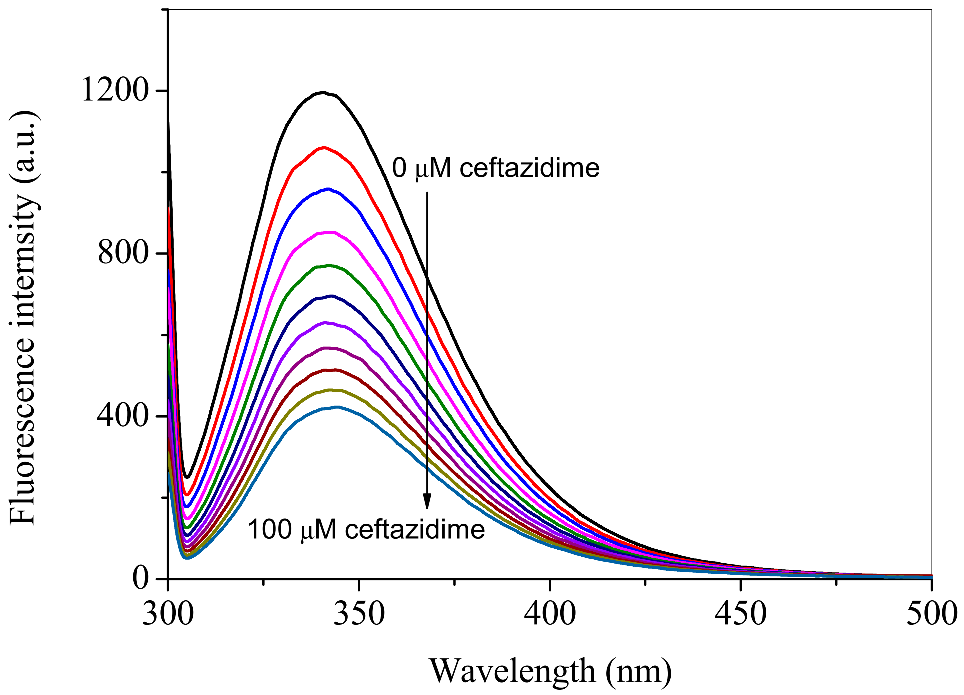

Figure S8. Observed fluorescence spectra of HSA in presence of several concentrations of ceftazidime ranging from 0 to 100 μM with a constant increment of 10 μM at the excitation wavelength of 295 nm at 25 °C in presence of several concentrations of 150 μM paracetamol.

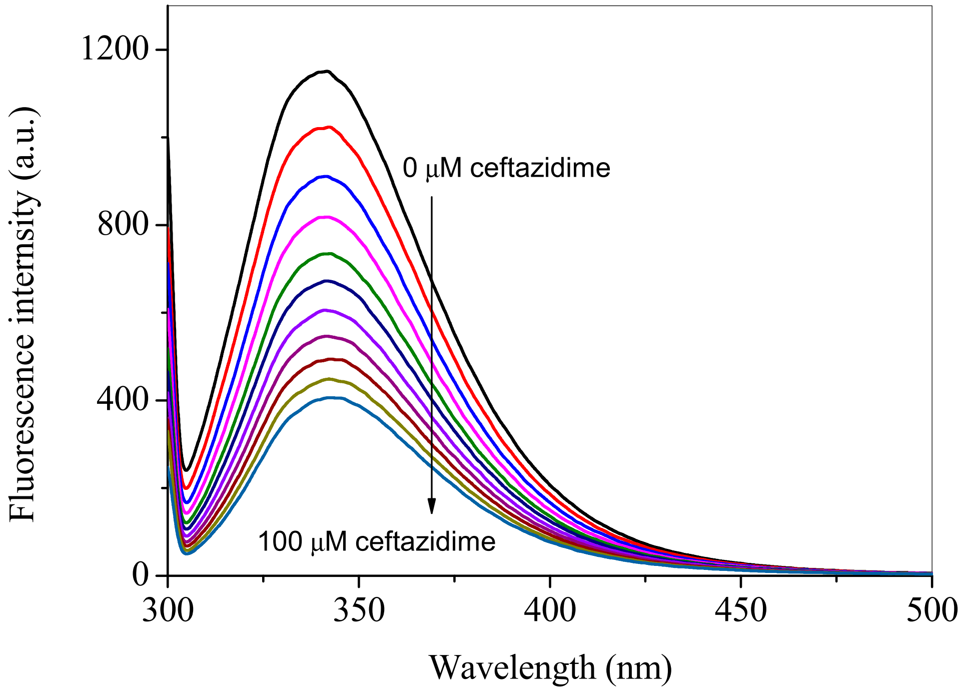

Figure S9. Observed fluorescence spectra of HSA in presence of several concentrations of ceftazidime ranging from 0 to 100 μM with a constant increment of 10 μM at the excitation wavelength of 295 nm at 25 °C in presence of several concentrations of 250 μM paracetamol.

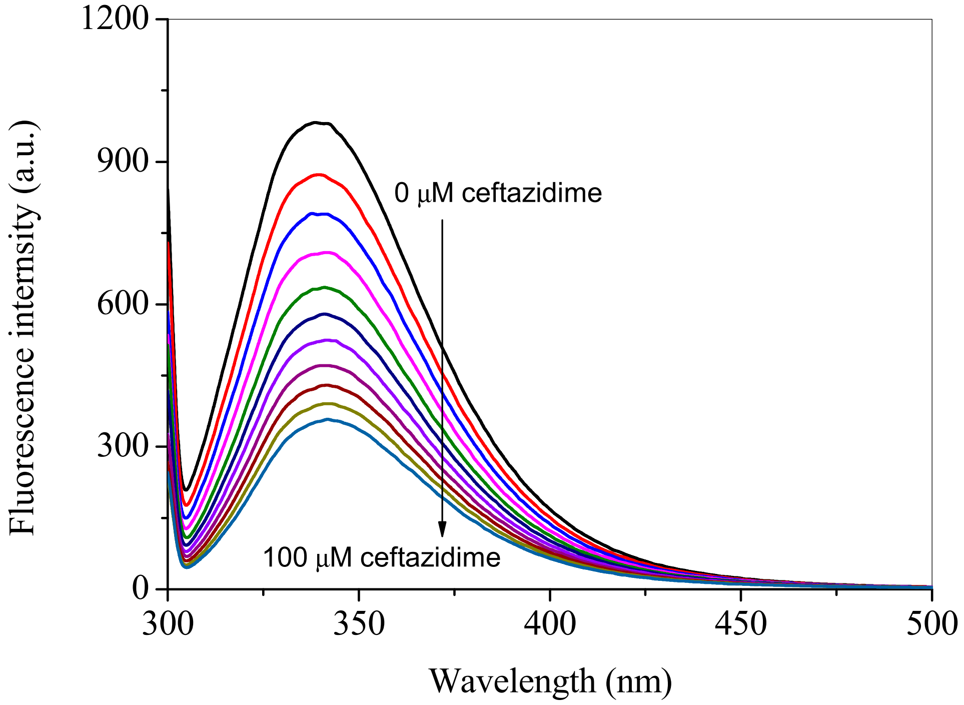

Figure S10. Observed fluorescence spectra of HSA in presence of several concentrations of ceftazidime ranging from 0 to 100 µM with a constant increment of 10 µM at the excitation wavelength of 295 nm at 25 °C in presence of several concentrations of 500 µM paracetamol.

Table S1. Lineweaver–Burk constants of the interaction of HSA with ceftazidime in presence of several concentrations of paracetamol.

[paracetamol] (μM)	K_{LB} (M^{-1})
0	6.1×10^3
50	5.7×10^3
100	5.6×10^3
150	4.8×10^3
250	4.5×10^3
500	4.3×10^3

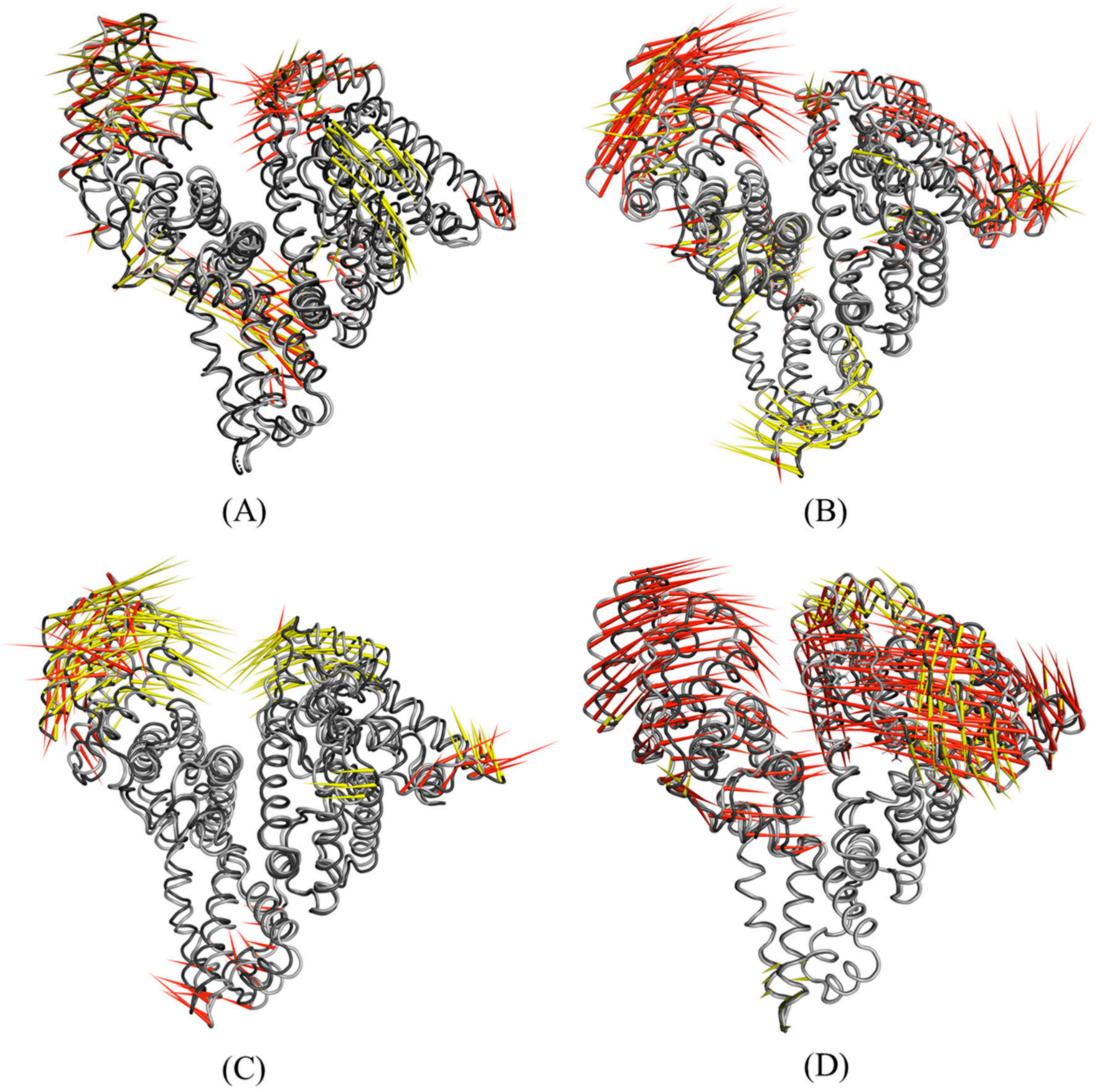

Figure S11. Porcupine analysis for (A) HSA, (B) Paracetamol-HSA complex, (C) Ceftazidime-HSA complex, (D) Paracetamol-Ceftazidime-HSA complex. Red spikes represent PC1 and yellow spikes represents PC2.

[1] G.M. Morris, R. Huey, W. Lindstrom, M.F. Sanner, R.K. Belew, D.S. Goodsell, A.J. Olson, AutoDock4 and AutoDockTools4: Automated docking with selective receptor flexibility, 30 (2009) 2785-2791.
[2] D. Van Der Spoel, E. Lindahl, B. Hess, G. Groenhof, A.E. Mark, H.J.C. Berendsen, GROMACS: Fast, flexible, and free, 26 (2005) 1701-1718.
[3] V. Zoete, M.A. Cuendet, A. Grosdidier, O. Michielin, SwissParam: A fast force field generation tool for small organic molecules, 32 (2011) 2359-2368.
[4] R. Kumari, R. Kumar, A. Lynn, g_mmpbsa—A GROMACS Tool for High-Throughput MM-PBSA Calculations, Journal of Chemical Information and Modeling, 54 (2014) 1951-1962.
